# Development and validation of COMPASS: clinical evidence of orphan medicinal products – an assessment tool

**DOI:** 10.1186/1750-1172-8-157

**Published:** 2013-10-09

**Authors:** Eline Picavet, David Cassiman, Bert Aertgeerts, Steven Simoens

**Affiliations:** 1 Department of Pharmaceutical and Pharmacological Sciences, KU Leuven, Herestraat 49, PO box 521, Leuven 3000, Belgium; 2 Department of Hepatology, University Hospital Leuven, Herestraat 49, Leuven 3000, Belgium; 3 Department of General Practice, KU Leuven, Kapucijnenvoer 33, PO box 7001, Leuven 3000, Belgium; 4 Belgian Centre for Evidence-Based Medicine, Leuven, Belgium

**Keywords:** Quality assessment, Quality assessment tool, Quality of reporting

## Abstract

**Background:**

Rare diseases are defined as life-threatening or chronically debilitating diseases with a prevalence of 50 out of 100,000 individuals or less. Orphan medicinal products (OMPs) are intended for the treatment of rare diseases. The assessment of quality of evidence in small populations is often complex. Many generic tools are unfit. Therefore, the aim of this study was to develop and validate a new tool to assess the quality of OMPs' clinical evidence (COMPASS).

**Methods:**

Firstly, a draft version of the COMPASS tool, developed by the authors and consisting of three parts, was amended based on suggestions obtained in four rounds of expert consultation. Secondly, the tool was put through three rounds of validation. The data source was information provided on the Orphanet website and in European Public Assessment Report (EPAR) document of the European Medicines Agency.

**Results:**

The first pilot round revealed a high (92.2%) inter-rater agreement for part one of the tool. After further improvements, the final inter-rater agreement was 86.4% for part two (on methodological quality) and three (on quality of reporting) of the tool. The COMPASS tool does not attempt to score or rank the quality of clinical evidence, but rather to give an outline of various, key elements with respect to quality of clinical evidence of OMP studies.

**Conclusions:**

The COMPASS tool can be applied to assess the quality of evidence of an OMP based on information in the registration dossier, for example by local reimbursement agencies, pharmacists or clinicians. In that way, the tool can contribute to making reimbursement and/or treatment decisions increasingly more founded on the principles of evidence-based decision making.

## Background

Rare diseases are defined as life-threatening or chronically debilitating diseases with a prevalence of 50 out of 100,000 individuals or less [1]. It is estimated that there are currently between 5,000 and 7,000 rare diseases [2]. Orphan medicinal products (OMPs) are intended for the treatment of rare diseases [1]. Studies to evaluate the effect of an OMP in patients with rare diseases are often hampered by the difficulty of enrolling a sufficient number of patients [3,4]. For example, N-acetylglutamate synthase (NAGS) deficiency is a very rare disorder that can cause neonatal life-threatening hyperammonemia. To date, only few patients with NAGS deficiency have been identified. In Europe, treatment with carglumic acid for NAGS deficiency has been authorized based on efficacy data from four case reports and 12 patients in a retrospective data collection study [5,6]. For some OMPs, it is clear that the *quantity* of clinical evidence cannot be obtained. Even so, achieving the highest quality of evidence should still be aimed for [7].

But how do we define quality of clinical evidence? According to GRADE (Grading of Recommendations Assessment, Development and Evaluation), quality of evidence reflects the extent of our confidence that the estimates of an effect are correct [8]. Traditionally, randomized controlled studies are regarded as the gold standard in achieving high quality of evidence whereas case controls or case studies are considered of lesser, but not lacking, value [7,9,10]. Nevertheless, high quality evidence is needed to guide clinical decision-making [11].

The assessment of quality of evidence in small populations at the time of registration and/or reimbursement is often complex. However, the rising number of authorized OMPs and their increased use in clinical practice emphasizes the need for an objective assessment. Quality assessment involves evaluation of a study’s validity, i.e. the degree to which its design, conduct and analysis have minimised biases or errors [12]. In general, there are three ways to assess the quality of studies: individual markers, checklists and scales [13-15]. Many generic tools, from simple checklists to extensive questionnaires, are currently used to assess studies [12,16]. However, the majority of these instruments are unfit to assess clinical studies of OMPs, as they do not take into consideration the difficulties (ie small sample size, use of surrogate endpoints, etc.…) that are inextricably bound up with these studies. According to Khan, new tools can be developed, keeping in mind that all components of the tool should be selected with due consideration for its purpose. These components should capture both generic methodological issues and issues specific to the subject under review [12].

Therefore, the aim of this study was to develop and validate a new tool, COMPASS (Clinical evidence of Orphan Medicinal Products – an ASSessment tool), to assess the quality of OMPs clinical evidence that is presented for OMPs at the time of marketing authorization in the EU.

## Methods

### Design of the tool

The design of the tool was conceptualised after consulting the Centre for Evidence-Based Medicine (CEBAM), Leuven, Belgium. A draft version of the tool was drawn up based on elements derived from existing checklists supplemented with items specifically related to rare diseases and OMPs [13-15].

### Validity of the COMPASS tool

The draft version of the tool was proofread by two laymen to increase readability. Subsequently, four expert consultations were organised with six experts (in a two-two-one-one fashion) with a view to increasing content validity (Table 1). All consultations were audio-taped and transcribed *verbatim*. The transcripts were analyzed in three steps. The first step was aimed at familiarizing with the data by reading and re-reading the transcripts. Secondly, a framework of key issues was identified. Finally, all issues were grouped according to the framework and interpreted. The draft version of the tool was adapted in accordance with all relevant issues, as deemed upon consensus by the researchers, raised at all consultations.

**Table 1:** Overview of expert consultations

		**Background**
**Expert consultation #1**		
	E.P.	Academic
	S.S.	Academic
	Expert 1	Physician - Regulatory
	Expert 2	Academic - Regulatory
**Expert consultation #2**		
	E.P.	Academic
	S.S.	Academic
	D.C.	Academic - Physician
	Expert 3	Pharmaceutical industry
	Expert 4	Hospital pharmacist
**Expert consultation #3**		
	E.P.	Academic
	Expert 5	Academic
**Expert consultation #4**		
	E.P.	Academic
	Expert 6	Academic - Regulatory

### Data source

The data source of the tool consisted only of information provided on the Orphanet website and in European Public Assessment Report (EPAR) and/or the Scientific Discussion (SD) document prepared by the Committee for Human Medicinal Products (CHMP) of the European Medicines Agency (EMA). These documents provide information about chemical, pharmaceutical, biological, toxico-pharmacological and clinical aspects of a drug [5]. For practical and privacy reasons, we did not have access to the original documents submitted to EMA. However, we anticipated that the publicly EPARs sufficiently reflect these original documents. The assessment of the methodological quality was restricted to studies that were described as 'pivotal’ or 'main’ clinical studies. The analyses were performed per study, as opposed to per orphan medicinal product, due to possible methodological differences between the studies. No additional data from publications of those studies was used due to possible unsystematic reporting and publication bias.

### Consistency of the COMPASS tool

A first pilot round was undertaken, in which five randomly selected OMPs (i.e. one from each “type”: betaine anhydrous (one indication, only literature reports), histamine dihydrochloride (one indication, open label study), idursulfase (one indication, RCT), pirfenidone (one indication, two RCTs) and sorafenib (two indications, two RCTs)) were analysed by two raters (E.P. and S.S.). The two raters completed all three parts of the COMPASS tool. A second pilot round was undertaken, in which four alphabetically successive OMPs (ziconotide, agalsidase alfa, sildenafil citrate and lenalidomide) were analysed by two raters (E.P. and D.C.). One rater completed all three parts of the tool, whereas the other completed the second and third part. The final version (Additional file 1) of the COMPASS tool was drawn up in accordance with the issues raised during the two pilot rounds.

The COMPASS tool consists of three parts; the first part collects general descriptive information about the OMP and its marketing authorization. The second part focuses on the assessment of the methodological quality (i.e. specifically related to study design, patient and study population, control arm, blinding, randomization and allocation, outcomes, adherence and statistical analysis) of the study. The last part assesses the quality of reporting. The Orphanet website was consulted to provide information on the prevalence of the rare disease in which the indication is authorized and its therapeutic need [2]. The registration of the pivotal studies on EudraCT and/or clinicaltrials.gov was evaluated on their respective websites [17,18].

In a third and final round, two raters (E.P. and S.S.) completed the tool for a sample of OMPs (n = 29). One rater (E.P.) completed all three parts of the tool, whereas the other (S.S.) completed the second and third part. Additionally, expertise in the medical field was believed necessary to answer the question “Is the duration of the study relevant to the natural history of the disease?”. Therefore, a third rater (D.C.) (ie a trained physician) also answered this question for all 29 OMPs. Upon disagreement between the raters, the assessment of D.C. was considered decisive.

In all three rounds, raters completed the tool independently and once-only. Additionally, raters were blinded with respect to results of others. The same information was available to all raters. After data collection, E.P. was responsible for comparison of the results.

### Analysis

All analyses (including the calculation of inter-rater agreement, by percent agreement calculation) were performed using MS Office Excel 2010.

## Results

### Validity of the COMPASS tool

During the expert consultations, a number of issues were discussed related to both the design (for example, on the sub-classification of the tool into three parts) and to the content of the tool (for example, on how to define a *valid* method of randomization). The total number of issues discussed per consultation is shown in Table 2. All relevant suggestions were implemented.

**Table 2:** Expert consultation - number of issues discussed

	**Number of issues discussed**
Expert consultation #1	44
Expert consultation #2	53
Expert consultation #3	48
Expert consultation #4	42

### Consistency of the COMPASS tool

The inter-rater agreement rates of the three rounds are shown in Table 3. In the first round, the overall inter-rater agreement was 87.1%. Also, there were small anomalies (ie when one rater answered 'No’ and the other answered 'Not reported’) for 3.7% of the answers. There was a slight increase in inter-rater agreement in the second round. Additionally, there was less (1.6% of the answers) confusion between 'No’ and 'Not reported’. However, this rate increased to 6.2% in the third round. In all rounds, disagreements between the raters were able to be resolved upon reviewing the data.

**Table 3:** Inter-rater agreement

	**Inter-rater agreement (%)**			
	**Overall**	**Part 1**	**Part 2**	**Part 3**
1st pilot round (n = 5)	87.1	92.2	76.9	
2nd pilot round (n = 4)	NA	NA	77.5	
3rd round (n = 29)	NA	NA	86.4	

*n*, number of orphan medicinal products under review; *NA*, not applicable.

All three raters independently evaluated the relevance of the study duration with respect to the natural history of the disease. There was agreement between three raters for 65.4% of the studies. In case of disagreement, the rater with a medical background was more inclined to assess the study duration as appropriate (77.8%) than raters without a medical background (E.P. 27.8% and S.S. 22.2%). Additionally, the raters without a medical background experienced more difficulties in evaluating study duration, as shown by their choice of “Don’t know” respectively nine and two times.

## Discussion

The goal of this research was to develop and validate a new tool COMPASS to assess the quality of OMPs clinical evidence presented at the time of marketing authorization in the EU. The COMPASS tool (Additional file 1) does not attempt to score or rank the quality of clinical evidence, but rather to give an outline of various, key elements with respect to quality of clinical evidence, as seen by experts. Ultimately, it is up to the evaluator to define minimum conditions of quality for an individual OMP or a set of similar OMPs. Ideally, these conditions should be defined without taking into account unmet need and disease severity, as these are not determining factors for quality of clinical evidence.

This COMPASS tool can be applied to assess the quality of evidence of an OMP based on the information in the registrations dossiers, for example by local reimbursement agencies for the review of clinical evidence or by pharmacists and clinicians upon considering a (new) treatment. Registration and reimbursement bodies currently acknowledge the data limitations for OMPs by providing them special considerations [19]. However, over time they are likely to become more sensitive to data requirements [20]. For example, non-binding recommendations for the approval of cancer drugs and biologics were issued by the Food and Drug Administration (FDA) several years ago [21]. Nowadays, deviations from the guidelines, stated in a similar European document, should be thoroughly justified [22].

To improve the reliability of results, data extraction should be performed independently by at least two raters. The reliability of results has shown to be independent of blinding, for that reason data extraction should not necessarily be blinded [12]. An exception on the number of raters can be made for the first part of the tool, as it collects more general and descriptive information about the OMP. Yet, minor differences between the raters can occur depending on the information source. To reduce variability, the source of information was pre-specified for some queries (ie specified the source of prevalence data to EPAR and Orphanet). There are more subjective questions in part two of the tool, emphasizing the need for independent raters.

Study quality is dependent, not only on methodological quality, but also on the quality of reporting [14]. Indeed, shortcomings in the reporting can complicate the interpretation of the methodological quality. Correct and complete information should provide the reader with the ability to make informed judgements about the validity of a study [23]. In practice, it was (nearly) impossible to assess the methodological quality of those (few) studies that were only very briefly discussed in the EPAR. Additionally, the interpretation was also complicated for those EPARs that consisted only of literature studies. To address the issue of poor reporting, part three of the tool focuses on quality of reporting. Additionally, for some questions, an additional check box was provided with 'Not reported’. Whilst in some cases the difference between answering 'No’ or 'Not reported’ may be vague, the subtle difference influences the evaluation of the quality of reporting.

The COMPASS tool has several strengths and weaknesses. The tool was developed after iterative rounds of expert consultation and underwent several pilot rounds to increase its validity. The tool assesses the level of clinical evidence that is presented in the pivotal studies at the time of marketing authorization. As such, it does not take into consideration the evidence in any of the supporting studies or evidence generated as part of post-marketing commitments. Adding data from publications would allow for quality control of the EPAR data, but was considered outside the scope of this study. Also, the tool is dependent on the quality of reporting in the EPAR and/or SD documents. Finally, a general medical knowledge of the rater is advisable to complete the tool. Additional check boxes ('Don’t know’ and 'Not reported’) were provided, to account for possible problems related to these last issues.

## Conclusions

In conclusion, we developed and validated a new tool for the assessment of clinical evidence of OMPs. The COMPASS tool can for example be used by local reimbursement agencies for the review of clinical evidence from OMP registration dossiers or by clinicians and pharmacists upon considering a (new) treatment. Furthermore, we hope that the COMPASS tool can initiate and add to the open debate on study standards for orphan medicinal products. In that way, the COMPASS tool can contribute to making reimbursement decisions increasingly more founded on the principles of evidence-based decision making.

## Abbreviations

COMPASS: Clinical evidence of orphan medicinal products – an assessment tool; OMP: Orphan medicinal product; EPAR: European public assessment report; FDA: Food and drug administration; SD: Scientific discussion; CHMP: Committee for human medicinal products; EMA: European medicines agency.

## Competing interests

The authors declare that they have no competing interests. No sources of funding were used to assist in the preparation of this manuscript.

## Authors’ contributions

The work presented was carried out in collaboration between all authors. EP, SS, DC and BA were involved in the initiation of the project and the design of the methods. EP, SS and DC actively participated in the expert consultations and in the data collection. EP analysed the data and interpreted the results. EP, SS, DC and BA discussed the results. EP and SS wrote the draft paper. SS, DC and BA revised the draft paper. All authors have read and approved the final manuscript.

## Supplementary Material

Additional file 1 COMPASS tool. Click here for file

## References

[B1] Commission E Regulation (EC) No 141/2000 of the European parliament and of the council of 16 December 1999 on orphan medicinal products Off J Euro Comm 2000 L18 1 5

[B2] Orphanet - The portal for rare diseases and orphan drugshttp://www.orpha.net PMC3669585 23761721

[B3] Wilcken B Rare diseases and the assessment of intervention: what sorts of clinical trials can we use? J Inherit Metab Dis 2001 24 291 298 10.1023/A:1010387522195 11405347

[B4] Tambuyzer E Rare diseases, orphan drugs and their regulation: questions and misconceptions Nat Rev Drug Discov 2010 9 921 929 10.1038/nrd3275 21060315

[B5] European Medicines Agencyhttp://www.ema.europa.eu/ema/

[B6] Haberle J Role of carglumic acid in the treatment of acute hyperammonemia due to N-acetylglutamate synthase deficiency Ther Clin Risk Manag 2011 7 327 332 21941437 10.2147/TCRM.S12703 PMC3176164

[B7] Day S Evidence-based medicine and rare diseases Rare Dis Epidemiol 2010 686 41 53 10.1007/978-90-481-9485-8_3 20824438

[B8] Balshem H Helfand M Schunemann HJ Oxman AD Kunz R Brozek J Vist GE Falck-Ytter Y Meerpohl J Norris S Guyatt GH GRADE guidelines: 3. Rating the quality of evidence J Clin Epidemiol 2011 64 401 406 10.1016/j.jclinepi.2010.07.015 21208779

[B9] Barton S Which clinical studies provide the best evidence? The best RCT still trumps the best observational study BMJ 2000 321 255 256 10.1136/bmj.321.7256.255 10915111 PMC1118259

[B10] Griggs RC Batshaw M Dunkle M Gopal-Srivastava R Kaye E Krischer J Nguyen T Paulus K Merkel PA Clinical research for rare disease: opportunities, challenges, and solutions Mol Genet Metab 2009 96 20 26 10.1016/j.ymgme.2008.10.003 19013090 PMC3134795

[B11] Kruer MC Steiner RD The role of evidence-based medicine and clinical trials in rare genetic disorders Clin Genet 2008 74 197 207 10.1111/j.1399-0004.2008.01041.x 18657147

[B12] Undertaking systematic reviews of research on effectiveness http://www.york.ac.uk/inst/crd/pdf/Systematic_Reviews.pdf

[B13] Jadad AR Moore RA Carroll D Jenkinson C Reynolds DJ Gavaghan DJ McQuay HJ Assessing the quality of reports of randomized clinical trials: is blinding necessary? Control Clin Trials 1996 17 1 12 10.1016/0197-2456(95)00134-4 8721797

[B14] Deeks JJ Dinnes J D'Amico R Sowden AJ Sakarovitch C Song F Petticrew M Altman DG Evaluating non-randomised intervention studies Health Technol Assess 2003 7 iii-173 10.3310/hta7270 14499048

[B15] Dechartres A Charles P Hopewell S Ravaud P Altman DG Reviews assessing the quality or the reporting of randomized controlled trials are increasing over time but raised questions about how quality is assessed J Clin Epidemiol 2011 64 136 144 10.1016/j.jclinepi.2010.04.015 20705426

[B16] Olivo SA Macedo LG Gadotti IC Fuentes J Stanton T Magee DJ Scales to assess the quality of randomized controlled trials: a systematic review Phys Ther 2008 88 156 175 10.2522/ptj.20070147 18073267

[B17] EUdraCThttp://eudract.ema.europa.eu/

[B18] ClinicalTrials.govclinicaltrials.gov/

[B19] Heron L Laurenson S Costello J Anderl C Knospe J Gaining reimbursement of orphan products in Europe: challenges Due to wide variations in evidence requirements and processes Value in Health 2012 15 A309

[B20] Jeffery M White R Orphan drug pricing and access - current situation and future trends in Eu Value in Health 2012 15 A309

[B21] Food and Drug Administration Guidance for Industry: Clinical Trial Endpoints for the Approval of Cancer Drugs and Biologics http://www.fda.gov/downloads/Drugs/GuidanceComplianceRegulatoryInformation/Guidances/ucm071590.pdf

[B22] European Medicines Agency Guideline on the evaluation of anticancer medicinal products in man http://www.ema.europa.eu/docs/en_GB/document_library/Scientific_guideline/2013/01/WC500137128.pdf

[B23] Begg C Cho M Eastwood S Horton R Moher D Olkin I Pitkin R Rennie D Schulz KF Simel D Stroup DF Improving the quality of reporting of randomized controlled trials - The CONSORT statement JAMA 1996 276 637 639 10.1001/jama.1996.03540080059030 8773637

