# Supplementary material for: Development and validation of COMPASS: clinical evidence of orphan medicinal products – an assessment tool
Source: Orphanet J Rare Dis. 2013 Oct 9;8:157. doi: 10.1186/1750-1172-8-157 (PMC3856624; doi:10.1186/1750-1172-8-157)
Supplement: Additional file 1 — COMPASS tool. [file 1750-1172-8-157-S1.docx]

# Supplemental file

COMPASS tool

(following pages)

**COMPASS**

**Clinical evidence of Orphan Medicinal Products – an ASSessment tool**

__________________________________________________________________________

**Introduction**

This COMPASS tool is designed to collect data included in registration dossiers of orphan medicinal products submitted to the European Medicines Agency. Data sources include the European Public Assessment Report (EPAR) and the Scientific Discussion (SD) document prepared by the Committee for Human Medicinal Products of the European Medicines Agency. The tool consists of three parts; the first part collects general descriptive information about the orphan medicinal product and the registration dossier. The second part focuses on the assessment of the methodological quality of the pivotal clinical study(ies). The last part assesses the quality of reporting.

**Tracking information**

Orphan medicinal product (INN) ..........

Reviewer ..........

Date(s) of the review ..........

**Part 1: Descriptive information**

*Complete section 1.1* ***for each orphan medicinal product****.*

- 1. ***Marketing authorization***

INN ..........

ATC code ..........

Marketing authorization holder ..........

Date of orphan designation (first condition) ..........

Date of marketing authorization (first indication) ..........

Number of centrally authorized indications

- Orphan indications ..........
- Non-orphan indication ..........

Type of marketing authorization (initial type)

Normal  Conditional  Exceptional circumstances

Protocol assistance requested

Yes  No  *Not reported*

*Complete section 1.2 to 1.4* ***for each orphan indication****.*

- 1. ***Indication***

What is the indication for which the OMP is authorized? ..........

What is the therapeutic area?

Infectious disorders  Oncology  Endocrine and metabolic disorders

Neurologic and psychiatric disorders  Other; ……….

What is the prevalence of rare disease in which the indication is authorized?

According to Orphanet: …...….  *Not reported*

According to the EPAR: ……….  *Not reported*

- 1. ***Dose finding***

Were dose finding studies performed?

Yes  No  *Not reported*

- 1. ***Therapeutic need***

The indication is:  life-threatening,  chronic (≥ 6m), and/or  seriously debilitating

The OMP was approved on the grounds of  significant benefit or  no approved alternative

What were the available (approved) alternatives? ………. Information source: ……….

*Complete section 1.4, 1.5, Part II and Part III* ***for each pivotal study*****.*

* ‘pivotal’ or ‘main’ study, as defined in the EPAR

- 1. ***Clinical study characteristics (pivotal study)***

Study identification number: ……….

Primary study objective: ……….  *Not reported*

Study phase: ……….  *Not reported*

Study design type: ……….

Blinding  No  Single  Double  Triple

Randomization  No  Yes

Control intervention  Placebo  Standard of care  Other: ……….

Adaptive study design (specify)? ……….  No

Mono- or multinational study?  Mono-national  Multinational  *Not reported*

Mono- or multicentre study?  Mono-centre  Multicentre  *Not reported*

How many individuals were included? ……….  *Not reported*

What were the primary endpoints? ……….  *Not reported*

What were the secondary endpoints? ……….  *Not reported*

- 1. ***Dissemination and registration***

Have the results of this pivotal study been published* in a peer-reviewed international journal? (* prior to and after registration)  No  Yes; specify (Author, Journal, year): ……….

Has the study been registered on

- EudraCT?  No  Yes, on ../../…. (date)
- clinicaltrials.gov?  No  Yes, on ../../…. (date)

Was the study protocol a priory approved by an Ethics Committee? (“carried out in accordance with the ethical standards of Directive 2001/20/EC”)

Yes  No  *Not reported*

Additional comments with respect to part 1: ……….

**Part 2: Analysis of study quality**

**Study design**

According to a standard study design algorithm, what is the study design? ..........

Did ethical considerations play a role in the choice of study design? (i.e was it reported as such?)

Yes, specify; ………  No  *Not reported*

Did practical considerations play a role in the choice of study design? (ie was it reported as such?)

Yes, specify; ………  No  *Not reported*

**Patient and study population**

Does the study population represent the patient population? (ie represent the possible heterogeneity in the patient population)

Yes  No, because ……….  Don’t know

Were any a priori power calculations performed, to test whether the study size is sufficient to test a clinically significant difference? (“power of a study” is defined a s the probability of reaching a true positive conclusion)

Yes, and the required number of inclusions was achieve

Yes, but the required number of inclusions was not achieved

Yes, but it is unclear if the required number of inclusions was achieved

No  Not reported

**Control arm**  N/A due to study design

What type of control arm was used?

Active comparator  Placebo  Historical or published data

Other, specify; ……….

Were the different groups similar at baseline regarding the most important prognostic indicators*? (* as defined in the EPAR)

Yes (statistically determined)  Likely (not statistically determined)  *Not reported*

No (statistically determined)  Unlikely (not statistically determined)

**Blinding**  N/A due to study design

Was the outcome assessor blinded?

Yes  No  No, but justified  *Not reported*

Was the care provider blinded?

Yes  No  No, but justified  *Not reported*

Was the patient blinded?

Yes  No  No, but justified  *Not reported*

**Randomization and allocation**  N/A due to study design

Have patients been allocated to different groups?

Yes, randomly  Yes  No  *Not reported*

Is the method of randomization valid? (Valid methods are 1) central randomization (remote from the patient recruitment centre); 2) sequentially numbered drug containers (prepared by an independent pharmacy, identical containers). Invalid methods are 1) open random number tables; 2) pseudo-random methods based on for example date of birth, postal code; 3) sequentially numbered opaque and sealed envelopes)

Yes  No  *Not reported*

**Endpoints**

Was a Quality-of-Life related endpoint included to determine the impact of the OMP on the quality of life of the patients?

Yes, using a generic scale  Yes, using a disease-specific scale

Yes, both  No, neither  *Not reported*

According to those scales, was an improvement in QoL (statistically) observed?

Yes, specify; ……….  No  NA

Is the duration of the study relevant to the natural history of the disease?

Yes  No  Don’t know

**Adherence**

Was there non-adherence to the study protocol? (ie major protocol violations, dispensing errors, ...)

Yes, by some patients  Yes, by the researcher(s)

Yes, both  No  *Not reported*

If so, specify: ……….

Has patient adherence to the therapy been assessed?

Yes  No  *Not reported*

If so, specify: ……….

**Statistical analysis**

Is the analysis appropriate to the hypothesis and to the data? (ie clarity of the statistical analysis; in case of multiple primary endpoints, with adjustments for multiple testing)

Yes  No  Don’t know

Are summary statistics provided?

No

Yes, at baseline for  all endpoints,  some endpoints or  only primary endpoints

Statistically tested (with p-values)?  Yes  No

Yes, at outcome for  all endpoints,  some endpoints or  only primary endpoints

Statistically tested (with p-values)?  Yes  No

Did the primary analysis include an intention-to-treat analysis? (ie everyone randomized is included in the final analysis and there are no selective dropouts)

Yes  No  NA  *Not reported*

What is reported with respect to patients lost to follow up (LTFU)?

Reasons for LTFU  Characteristics of LTFU patients  Both

Neither  NA

**Conclusions**

Are any conclusions drawn on the efficacy of the OMP in line with the results?

Yes  No  Don’t know  NA (no conclusions)

Additional comments with respect to part 2: ……….

**Part 3: Quality of reporting in the EPAR**

Were research hypotheses stated a priori?

Yes  No  *Not reported*

Are the main endpoints described? (explained, not just listed)

Yes  No

Are the sampling criteria described?

Yes  No  NA

Are the characteristics (ie age, gender …) of the study population described?

Yes  No

Are possible biases and/or potential confounders described?

Yes  No

Are the interventions described?

Yes  No  NA

Does the EPAR provide point estimates and measures of variability? For which endpoints?

Yes, point estimates;  for all endpoints  for some endpoints

Yes, measure of variability  for all endpoints  for some endpoints

No, neither

Have actual probability values been reported (unless the value is less than 0.001)?

Yes  No

Additional comments with respect to part 3: ……….
